# The Emerging Role of the Molecular Chaperone Clusterin in Parkinson’s Disease

**DOI:** 10.3390/ijms26136351

**Published:** 2025-07-01

**Authors:** Giulia Carini, Salihu Mohammed, Alice Filippini, Ileana Ramazzina, Isabella Russo

**Affiliations:** 1 Unit of Biology and Genetics, Department of Molecular and Translational Medicine, University of Brescia, Via Europa 11, 25123 Brescia, Italy; giulia.carini@unibs.it (G.C.); alice.filippini@unibs.it (A.F.); 2 Department of Medicine and Surgery, University of Parma, Via Gramsci 14, 43126 Parma, Italy; salihu.mohammed@unipr.it; 3 IRCCS Istituto Centro San Giovanni di Dio Fatebenefratelli, Via Pilastroni 4, 25125 Brescia, Italy; 4 Centre for Molecular and Translational Oncology (COMT), University of Parma, Parco Area delle Scienze 11/a, 43124 Parma, Italy; 5 Biostructures and Biosystems National Institute (INBB), Viale Medaglie d’Oro 305, 00136 Rome, Italy

**Keywords:** Clusterin, Parkinson’s disease, α-Synuclein, neurons, astrocytes

## Abstract

Clusterin (CLU) is a heterodimeric, ATP-independent molecular chaperone that exhibits high expression in the brain. While CLU primarily functions in the extracellular environment, its chaperone activity in the intracellular compartment under different stress conditions, as well as its involvement in various signaling networks, has been demonstrated. CLU has been extensively associated with Alzheimer’s Disease; however, increasing evidence links this chaperone to Parkinson’s Disease (PD) as well. Thus, in this review we will discuss evidence concerning the involvement of CLU in the pathogenesis of PD with a particular focus on molecular mechanisms leading to the formation and the spreading of alpha-Synuclein (α-Syn) aggregates. Specifically, the role of CLU will be discussed in neurons and in glial cells, taking into account that the neuron–glia cross-talk is an essential and dynamic interplay that is compromised in neurodegenerative disorders. Moreover, the possible role of CLU as a biomarker in different biological fluids, such as cerebrospinal fluid, plasma, and serum, and its therapeutic potential will be addressed. In this regard, the past years have seen huge efforts to discover molecules able to mitigate α-Syn burden and its related toxicity. Overall, this overview highlights CLU as an intriguing target that can affect biochemical events underlying PD pathology.

## 1. Introduction

Molecular chaperones, most of which belong to the Heat Shock Protein (Hsp) families, act in different branches of the proteostasis network to assure protein homeostasis. They are involved in the assistance of nascent proteins to acquire the proper folding and in counteracting and reverting misfolded and aggregated proteins, as well as driving the misfolded ones to the degradation systems [1]. Given their crucial role, it is not surprising that molecular chaperones are active in different subcellular compartments and in the extracellular milieu [1,2].

The impairment of protein homeostasis represents the key event in the so-called protein-misfolding diseases, including Parkinson’s Disease (PD) and Alzheimer’s Disease (AD), the most prevalent neurodegenerative disorders worldwide [3]. Accordingly, exploring the role of molecular chaperones in the onset and progression of these diseases can pave the way for the development of innovative molecular therapeutic approaches.

Of interest, Clusterin (CLU) is an intriguing molecular chaperone, as its activity is documented in both the extracellular and intracellular compartments. CLU has been extensively associated with AD; however, starting from the pioneering study of Sasaki and colleagues (2002) [4], it has also become an attractive target for PD. Based on these observations, this review will focus on the CLU chaperone activity in the formation and the clearance of alpha-Synuclein (α-Syn) toxic species, critical events in the pathogenesis of PD. Of note, we specifically discuss the role of CLU in both neurons and glia, since the “neuron-glia shuttle” plays a crucial role in healthy and pathological conditions.

### 1.1. Clusterin: Biogenesis, Distribution, and Chaperone Activity

**Figure 1:**
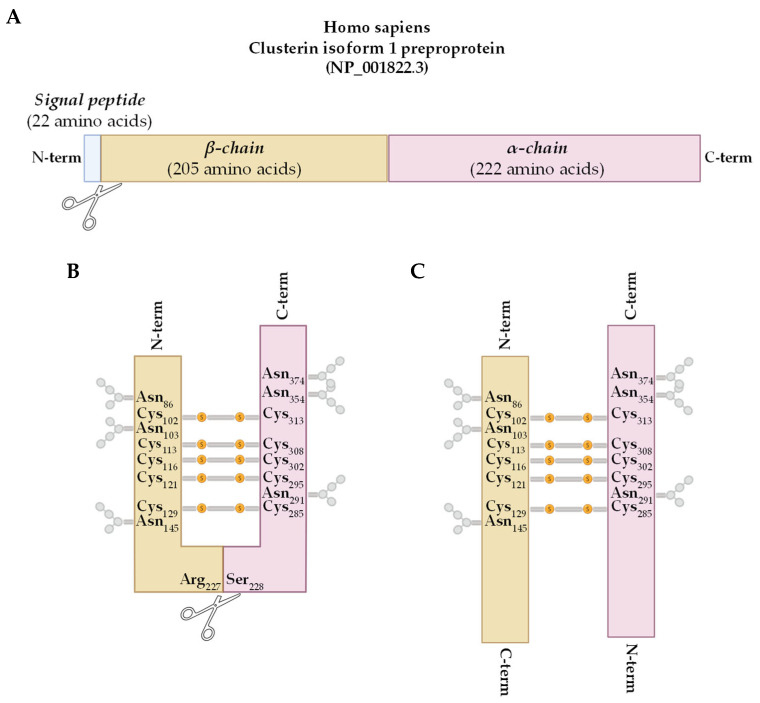
Schematic representation of the main events leading to the mature form of sCLU. (**A**) The N-terminal ER signal peptide is cleaved from the preproclusterin precursor (NP_001822), producing the CLU immature precursor form. (**B**) In the ER and the Golgi apparatus, the CLU precursor undergoes heavy glycosylation at six Asparagine (Asn) residues. (**C**) Finally, a proteolytic cleavage between residues Arginine 227 (Arg) and Serine 228 (Ser) in the Golgi apparatus generates the α (~40 kDa) and β (~35 kDa) chains, which are linked in an anti-parallel manner by five disulfide bonds (-s-s-). The biosynthetic pathway results in a mature α/β heterodimeric CLU protein (~75–80 kDa) that is secreted into the extracellular space.

In humans, the CLU gene (NG_027845.1) is situated at the 8p21.1 locus on the short arm of chromosome 8. It has 11 exons of different sizes, with 2 untranslated ones [5]. Starting at the canonical AUG in exon 2 of the CLU mRNA (NM_001831), an initial unfolded preproclusterin precursor (NP_001822) is translated. Then, a subsequent cleavage of the N-terminal endoplasmic reticulum (ER) signal peptide drives the amino acid chain to the ER for post-translational modifications (PTMs) such as phosphorylation and extensive glycosylation to generate an immature CLU precursor. It further undergoes heavy glycosylation and proteolytic cleavage between residues Arginine 227 and Serine 228 in the Golgi apparatus to generate the α (~40 kDa) and β (~35 kDa) chains. In an anti-parallel manner, five disulfide bonds reassemble the α and β chains, resulting in a mature 75–80 kDa α/β heterodimeric CLU protein that is secreted into the extracellular space (sCLU; Figure 1) [6,7,8]. The extensive PTMs have been shown to affect the stability, solubility, functions, and localization of the protein [9,10,11].

In addition to the well-characterized sCLU, other alternative isoforms have been reported, albeit with controversies regarding their biogenesis, localization, and physiological roles. These are known as Intracellular CLU (iCLU) which includes cytoplasmic CLU, nuclear CLU [12], and mitochondrial CLU [5]. iCLU could derive from an impaired secretory pathway, reuptake of sCLU after its extracellular release, and/or inadequate PTMs necessary for the generation of mature sCLU [11,13]. In this regard, several reports have indicated that CLU, both sCLU and iCLU, could exhibit non-canonical localization in response to different cellular stressors [14,15,16].

CLU is expressed in multiple organs and tissues. Wong and colleagues (1994) [17] demonstrated that human CLU total RNA expression progressively increased during gestation and into adulthood. Moreover, they also showed that the mRNA expression is relatively higher in the brain, pancreas, and liver as compared to other organs such as the heart, kidneys, and lungs [17]. CLU is also found in the reproductive organs [18] and in body fluids such as plasma and the cerebrospinal fluid (CSF) [19,20]. Interestingly, regarding the Central Nervous System (CNS), CLU is prominently and widely expressed by astrocytes, a subtype of glial cells [21,22], whereas its neuronal expression is more restricted to cortical and hippocampal neurons [5,23].

CLU, also known as apolipoprotein-J, was the first identified extracellular chaperone in mammals and is among the most extensively studied. Due to the presence of a heat shock element in its promoter region, CLU is upregulated following different types of cellular injuries and exhibits functions similar to small Hsp, such as Hsp27 and αβ-crystallin [8,24,25]. It is essential in several cellular pathways, including protein folding, lipid transport, cellular stress responses, apoptosis regulation, neuroprotection, and immune modulation [26]. Moreover, CLU is a key player in various pathological conditions, such as cancer, metabolic syndromes, inflammatory disorders, neurodegenerative diseases, and aging-related changes [13,27,28,29]. Unlike other chaperones, CLU functions in an ATP-independent manner [30]. This enables it to act as a holdase, binding to and stabilizing proteins in a folding-competent state, primarily preventing protein aggregation under cellular stress or in the extracellular space, where the ATP availability is less than inside the cell [30,31,32]. Moreover, it has been reported that glycosylated iCLU isoforms might have similar chaperone activity as sCLU [10]; however, more studies are necessary to fully elucidate the role of these iCLU isoforms both under physiological and pathological conditions.

### 1.2. Parkinson’s Disease: Clinical Presentation and Neuropathological Features

PD is a prevalent neurodegenerative disorder with a complex etiology that involves multiple molecular mechanisms, as well as genetic and environmental factors. An intricate combination of these factors could likely contribute to PD and affect its course [33,34,35]. PD is characterized by the progressive degeneration of dopaminergic neurons in the *Substantia nigra pars compacta* and other areas of the brain, resulting in both motor and non-motor dysfunctions [36]. The cardinal motor symptoms of the pathology include bradykinesia, tremors, rigidity, and impairments in gait and balance, while the non-motor symptoms include neurobehavioral and cognitive deficits, sleep disorders, autonomic failures, and sensory impairments [36,37]. The pathological hallmark of PD is represented by the neuronal accumulation of the misfolded aggregated forms of α-Syn, called Lewy bodies (LBs) and Lewy neurites (LNs), first described by Frederic Lewy in 1912 [38,39,40]. α-Syn is a protein physiologically involved in neurotransmitter release, synaptic function, vesicular trafficking [41,42], and mitochondrial biogenesis [43,44]. However, in a pathological environment, α-Syn misfolds and aggregates in dimers, oligomers, and premature protofibrils that ultimately form amyloid fibrils [45,46]. Numerous factors can promote the aggregation of α-Syn [47]. In this regard, oxidative stress has been reported to directly contribute to α-Syn aggregation by inducing pathological modifications on α-Syn, such as tyrosine dimerization, nitrosylation, and methionine oxidation [48]. Of note, oxidative stress and neuroinflammation, another hallmark of PD [49,50], are interlinked pathophysiological processes, with one enhancing the other, resulting in a toxic feedback system [51]. Pathogenic α-Syn species can activate immune cells, such as microglia and astrocytes, triggering the release of inflammatory cytokines and increasing reactive oxygen and nitrogen species [49]. This process results in the amplification of oxidative stress and potentiates α-Syn aggregation and cell-to-cell propagation. Furthermore, chronic neuroinflammation can hamper protein clearance pathways, resulting in increased α-Syn aggregation and accumulation. Thus, inflammation could act as both a trigger and an amplifier of the pathology [52,53,54,55].

Interestingly, molecular chaperones such as Hsp27, Hsp40, Hsp70, and CLU have been associated with LBs and LNs in patients with PD [4,56]. Moreover, chaperones have shown a pivotal role in counteracting the multistep aggregation events of α-Syn, reducing the related neuronal dysfunction and the persistent inflammation [57,58,59]. Thus, targeting molecular chaperones could represent a prominent approach for developing efficient PD therapies.

## 2. The Role of CLU on the Aggregation of α-Syn

The functional and potential pathophysiological interaction between CLU and α-Syn was first demonstrated by Sasaki et al. (2002), highlighting the co-localization of CLU and α-Syn in LBs, indicating a potential role of CLU in intracellular α-Syn aggregation [4]. CLU was identified as one of the proteins associated with α-Syn aggregation even in mesenchymal stem cells with a rotenone-induced PD pathology [60]. Moreover, in our recent work we highlighted the role of CLU in the cellular defense mechanisms against α-Syn burden in SH-SY5Y neuroblastoma cells stably overexpressing α-Syn. Specifically, we showed that CLU upregulation is part of the biochemical response triggered by the cells to manage α-Syn overexpression. Remarkably, CLU expression was upregulated earlier and more robustly than other Hsp types, such as Hsp27, Hsp70, and Hsp90, involved in maintaining the protein homeostasis in several neurodegenerative diseases, including PD. In addition, of relevance, we provided evidence that the downregulation of CLU resulted in increased α-Syn aggregates within the cells [61]. In accord with our results, Yuste-Checa et al. (2021) reported that CLU delayed α-Syn aggregation and seeding activity in HEK293T and SH-SY5Y cells stably expressing GFP-α-Syn (A53T) [62].

Another piece of evidence of CLU and α-Syn interplay comes from another our study Filippini et al. (2021) [22], where we observed increased levels of CLU in mice injected with human α-Syn adeno-associated virus and with α-Syn Pre-Formed Fibrils (PFFs). These results suggest that CLU is upregulated in response to α-Syn aggregation, highlighting its potential role as a chaperone that can influence PD pathogenesis [22].

In addition to its well-known chaperone function, it has been shown that CLU interacts with the Very Low-Density Lipoprotein Receptor (VLDLR) and Apolipoprotein E Receptor 2 (ApoER2), triggering a Reelin-like pathway mediated by Dab1 [63]. Of note, recombinant Reelin treatment significantly reduced pathological intracellular α-Syn aggregates in differentiated SH-SY5Y cells exposed to α-Syn PFFs [64]. Altogether, this evidence suggests that CLU may attenuate α-Syn aggregation burden through its binding to ApoER2 and VLDLR receptors. However, this mechanism needs to be elucidated with further research.

Although there are still only a few papers that have demonstrated a link between CLU and PD, CLU has been shown to have a role in protein aggregation even in other neurodegenerative disorders. In a cell-free model of AD, Beeg et al. (2016) revealed that CLU binds Amyloid-β (Aβ)_1-42_ oligomers with high affinity and suppresses aggregation and fibril formation both in primary and secondary nucleation processes [65]. In addition, Wojtas et al. (2020) also demonstrated that recombinant CLU significantly hindered tau fibrilization in in vitro AD models [66]. Interestingly, while it has been shown that CLU delayed tau fibril elongation in a cell-free assay, it enhanced the seeding capacity of tau by stabilizing potent oligomeric species capable of initiating further aggregation in HEK293T and a primary mouse neuronal AD model [29,62]. However, in an in vivo mouse model of AD, the complete loss of CLU and its haploinsufficiency corresponded with a worsened condition of tau pathology and the substantial augmentation in amyloid load, respectively [66,67]. Analogously, CLU has been shown to interact with TAR DNA-Binding Protein 43 (TDP-43), which is associated with amyotrophic lateral sclerosis and frontotemporal degeneration. Specifically, in a mouse N2a neuroblastoma model, CLU inhibited TDP-43 aggregation and reduced the number of cytoplasmic inclusions [15]. Taken together, these observations indicate that CLU takes part in and can counteract the aggregation of amyloid proteins, including α-Syn. However, more investigations are required to clearly understand its implications, especially in in vivo systems.

## 3. The Role of CLU on the Clearance of α-Syn Aggregates by Glial Cells

α-Syn aggregates released from stressed or degenerating neurons might contribute to the propagation of pathological α-Syn species between neurons and throughout the brain [68,69]. Thus, the clearance mechanisms engaged by glial cells could be crucial to prevent the progression and spreading of pathological α-Syn and preserve the neuronal network and its functions [22,49,70,71,72,73,74]. In this context, molecular chaperones, including CLU, have been reported to play a crucial role in detoxifying and clearing α-Syn aggregates in astrocytic cells [22,75]. In our study, we showed that astrocytes take up the extracellular CLU/α-Syn PFFs complex through dynamin-dependent endocytosis [22]. Moreover, of particular interest, we demonstrated that CLU knock-out murine primary astrocytes and CLU knock-down human-induced Pluripotent Stem Cell (hiPSC)-derived astrocytes exhibited an increased internalization of α-Syn PFFs. These findings indicate that sCLU limits the uptake of extracellular α-Syn aggregates by astrocytes and suggest that this cellular pathway might contribute to α-Syn pathology and progression of PD [22]. Moreover, we recently found that astrocytic CLU levels are regulated by PD-related Leucine Rich Repeat Kinase 2 (LRRK2) through miR-22-5p [21]. In addition, we observed that astrocytes with an LRRK2 G2019S pathological mutation exhibited reduced ability to uptake α-Syn aggregates, and, of relevance, the treatment with miR-22-5p improves this ability [21]. Overall, our results indicate that the LRRK2-CLU pathway is involved in the internalization of α-Syn fibrils by astrocytes and that the modulation of this pathway might improve the clearance of extracellular α-Syn aggregates.

CLU has also been associated with the uptake of AD-related Aβ aggregates. In support of our results, it has been shown that CLU interferes with Aβ aggregates’ internalization by astrocytes [76,77]. In detail, human astrocytes isolated from post-mortem brain tissues showed a reduction in Aβ oligomer uptake after being exposed to Aβ oligomers/CLU complexes, suggesting that CLU might negatively affect Aβ aggregate internalization, potentially enhancing Aβ plaque formation. In contrast with these reports, it has been proposed that CLU has a positive effect on the spreading of pathological aggregates. Specifically, the downregulation of CLU in astrocytes reduces the internalization of Aβ oligomers, possibly leading to an increased amount of extracellular Aβ species and deposition [78]. Although CLU has been linked to amyloid protein uptake and clearance, its role is still not completely understood.

In support of the key role of the molecular chaperones on amyloid proteins, Sheehan and colleagues (2023) showed that BAG3, a macroautophagy chaperone, enhances the phagocytosis of α-Syn aggregates by astrocytes, preventing α-Syn pathology [75]. Moreover, Hsp27 and αβ-crystallin have been found to be increased in reactive astrocytes of PD patients with dementia [79] and expressed by reactive glial cells localized in proximity to senile plaques [80]. Taken together, these observations suggest an important interplay between chaperones and glial cells in the clearance of amyloid proteins to counteract the spreading of the pathology.

## 4. CLU as a Biomarker for PD

CLU has gained attention as a candidate biomarker for PD due to its multifaceted roles in protein aggregation, the clearance of amyloid proteins, oxidative stress, apoptosis, and neuroinflammation. Moreover, as a secreted glycoprotein, CLU can be indicative of both peripheral and CNS activity. Interestingly, as reported above, CLU has been detected in different biological fluids, including plasma, serum, and CSF [81]. Overall, these observations make CLU an accessible and non-invasive biomarker candidate for the diagnosis, monitoring, and progression of PD.

### 4.1. CLU in CSF

Because CSF directly reflects CNS processes, CLU levels in this fluid may provide insights into PD-related pathological mechanisms. However, research findings on CSF CLU levels in PD patients have been inconsistent. For instance, Vranová et al. (2010) found significantly higher concentrations of CSF CLU in PD patients compared to controls, particularly in individuals tested within two years of symptoms onset [82]. In the following years, Vranová and colleagues (2014) confirmed higher CSF CLU levels in PD and PD with dementia patients when compared to controls [83], and in a later study, compared to other neurodegenerative diseases such as dementia with LBs, AD, progressive supranuclear palsy, and multiple system atrophy [84]. Similarly, Maarouf et al. (2012) reported a twofold increase in postmortem ventricular CSF CLU in PD patients in comparison to controls [85]. Consistent with these findings, Paslawski and colleagues (2019, 2023) reported significantly higher CSF CLU levels in PD patients compared to controls in different cohorts [86,87]. Conversely, other studies have reported no correlation between CSF CLU levels and PD. Indeed, Lidström et al. (2001) found no significant differences in CSF CLU levels between PD patients and controls [88]. Similarly, van Dijk et al. (2013) reported no significant differences in CSF CLU levels between PD patients and healthy controls, nor any association with disease duration, stage, or severity [89]. In addition, Koníčková et al. (2023) found no significant differences in CSF CLU levels across various neurodegenerative diseases, including PD [90].

### 4.2. CLU in Blood

CLU has also been explored as a promising blood-based biomarker for PD. Several studies suggested that CLU levels in blood may reflect systemic inflammatory responses and oxidative stress, both key contributors to PD pathogenesis. Moreover, blood CLU levels have been correlated with disease severity and progression, highlighting its potential as a peripheral biomarker for tracking PD-related neurodegeneration. Specifically, Zhang et al. (2012) reported increased serum CLU levels in PD patients compared to controls [91]. However, Mnich et al. (2023) found no significant differences in serum CLU levels between PD patients and healthy controls in another cohort from Ireland [92]. The expression of CLU has also been detected in serum-derived exosomes. Jiang et al. (2019) found that exosomal CLU levels were significantly increased in PD patients, correlating with motor symptoms severity and cognitive decline, suggesting its utility in tracking disease progression [93]. However, another study did not find any significant differences in serum neuronal exosome CLU levels between PD patients and healthy controls [94,95].

Regarding plasma CLU levels, Polimeno et al. (2018) [96] reported elevated plasma CLU levels in younger PD patients (<60 years old) when compared to age-matched healthy controls, suggesting a potential role as an early biomarker. Additionally, an inverse correlation was observed between plasma CLU levels and cognitive performance (measured by the Mini-Mental State Examination), linking higher CLU levels to cognitive decline in PD [96]. Moreover, Lin et al. (2021) also reported significantly higher plasma CLU levels in PD patients compared to controls [97]. However, Kitamura et al. (2018) identified significantly decreased plasma exosomal CLU levels in PD patients when compared to healthy controls [98], and two other studies showed no significant differences between PD patients and healthy controls, nor any correlation with disease duration, stage, or severity [86,89].

The presence of CLU and its altered levels across CSF, blood, serum, and plasma emphasize its potential as a versatile biomarker for PD. While its accessibility in peripheral fluids offers advantages for non-invasive testing, its presence in CSF may provide a more direct reflection of CNS pathology. However, discrepancies across studies highlight the need for standardized methodologies, larger cohort studies, and longitudinal research to establish its clinical validity and specificity as a PD biomarker.

## 5. Conclusions: Can CLU Be a Therapeutic Target for PD?

CLU is emerging as a key player in PD pathogenesis and progression and thus could be a promising candidate for therapeutic interventions. One of the hallmark pathological features of PD is the aggregation of α-Syn, leading to the formation of LBs and LNs that contribute to neuronal toxicity and degeneration. As a molecular chaperone, CLU has been shown to reduce intracellular α-Syn aggregation, thereby mitigating its toxic effects on neurons [61,99]. Molecular chaperones, including CLU, play a crucial role in maintaining protein homeostasis by preventing the formation of toxic aggregates, assisting in protein refolding, and directing misfolded proteins toward degradation pathways such as the ubiquitin–proteasome system and the autophagy–lysosomal pathway [100,101]. Interestingly, therapeutic strategies aimed at enhancing chaperones’ expression or function have shown promise in reducing α-Syn accumulation in preclinical studies [102]. Regarding CLU, a recent study demonstrated that sCLU alleviated motor deficits and reduced midbrain dopamine neuron apoptosis in MPTP-induced PD mice by modulating autophagy [103]. In addition to its intraneuronal role, CLU may also influence α-Syn pathology by limiting the uptake of extracellular α-Syn aggregates by astrocytes [21,22], which could contribute to the spreading of α-Syn between neurons. While all these observations are supported by experimental evidence, their translational relevance remains hypothetical, and several aspects of CLU’s role in PD pathophysiology are still under active investigation.

**Figure 2:**
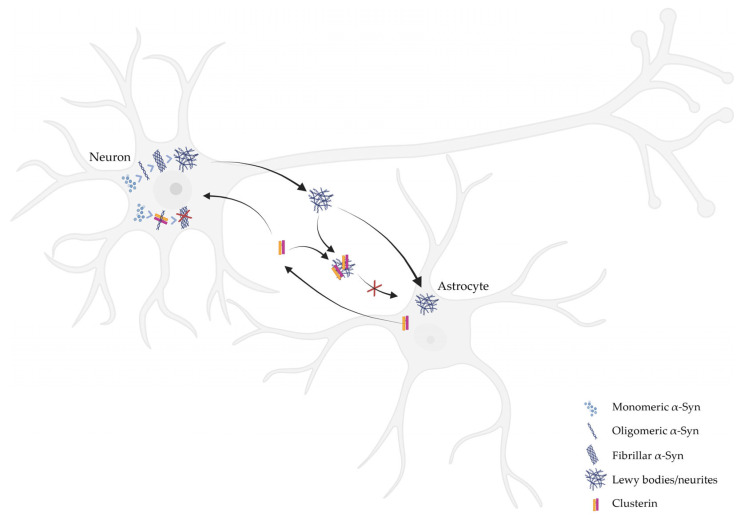
Role of CLU in neuronal α-Syn aggregation and uptake by astrocytes. In a pathological environment, α-Syn misfolds in neurons and aggregates into oligomeric and fibrillar forms, contributing to the formation of LBs and LNs. These misfolded α-Syn species are released into the extracellular space and can be taken up by neighboring astrocytes. CLU, which is mainly expressed and secreted by astrocytes, interacts extracellularly with pathological α-Syn aggregates. This interaction interferes with their uptake and clearance by astrocytes, potentially facilitating disease progression. Conversely, in neurons, CLU inhibits the pathological aggregation of α-Syn, suggesting a cell-dependent, dual role of CLU in modulating α-Syn pathology.

Overall, although CLU is clearly linked to PD, further research is essential to fully elucidate the role of CLU in the disease, as its effects appear to be cell-type-dependent. In neurons, CLU has been shown to reduce the pathological intracellular aggregation of α-Syn [61]; while in astrocytes, the presence of sCLU seems to impair the clearance mechanisms that normally help to eliminate toxic protein aggregates [21,22], thereby facilitating disease progression (Figure 2). This opposite role suggests that CLU may have both protective and detrimental effects, depending on the cellular environment. Thus, it is crucial to study the implications of CLU in in vivo systems, clarifying the interactions between different cell types in the brain. Understanding whether the protective effects in neurons outweigh the harmful effects in astrocytes—or vice versa—could provide important insights for therapeutic strategies targeting CLU in PD. Despite its potential as a therapeutic target, several challenges remain, and more investigations are required to clearly understand the implication of CLU in PD pathogenesis.

## Data Availability

Figures presented in this review were created with BioRender.com.

## Abbreviations

The following abbreviations are used in this manuscript.

ApoER2	Apolipoprotein E Receptor 2
α-Syn	Alpha-Synuclein
AD	Alzheimer’s Disease
Aβ	Amyloid-β
CNS	Central Nervous System
CSF	Cerebrospinal Fluid
CLU	Clusterin
ER	Endoplasmic Reticulum
Hsp	Heat Shock Protein
iCLU	Intracellular CLU
LRRK2	Leucine-Rich Repeat Kinase 2
LBs	Lewy Bodies
LNs	Lewy Neurites
PD	Parkinson’s Disease
PTMs	Post-Translational Modifications
PFFs	Pre-Formed Fibrils
sCLU	Secreted CLU
TDP-43	TAR DNA-Binding Protein 43
VLDLR	Very Low-Density Lipoprotein Receptor
